# The Patient Enablement Instrument-French version in a family practice setting: a reliability study

**DOI:** 10.1186/1471-2296-12-71

**Published:** 2011-07-07

**Authors:** Catherine Hudon, Martin Fortin, Francis Rossignol, Susie Bernier, Marie-Eve Poitras

**Affiliations:** 1 Département de médecine de famille, Université de Sherbrooke, Québec, Canada; 2 Centre de santé et de services sociaux de Chicoutimi, Québec, Canada; 3 Faculté de médecine et des sciences de la santé, Université de Sherbrooke, Québec, Canada

## Abstract

**Background:**

Patient enablement can be defined as the extent to which a patient is capable of understanding and coping with his or her health issues. This concept is linked to a number of health outcomes such as self-management of chronic diseases and quality of life. The Patient Enablement Instrument (PEI) was designed to measure this concept after a medical consultation. The instrument, in its original form and its translations into several languages, has proven to be reliable and valid. The purpose of this study was to evaluate the reliability of the French version of the PEI (PEI-Fv) in a family practice setting.

**Methods:**

One hundred and ten participants were recruited in a family medicine clinic in the Saguenay region of Quebec (Canada). The PEI-Fv was completed twice, immediately after consultation with a physician (T1) and 2 weeks after the consultation (T2). The internal consistency of the tool was assessed with Cronbach's α and test-retest reliability by intraclass correlation coefficient.

**Results:**

The mean score for the PEI-Fv was 5.06 ± 3.97 (95% confidence interval [CI]: 4.30-5.81) at T1 and 4.63 ± 3.90 (95% CI: 3.82-5.44) at T2. Cronbach's α was high at T1 (α_1 _= 0.93; 95% CI: 0.91-0.95) and T2 (α_2 _= 0.93; 95% CI: 0.91-0.95). The intraclass correlation coefficient was 0.62 (95% CI: 0.48-0.74), indicating a moderate test-retest reliability.

**Conclusions:**

The internal consistency of the PEI-Fv is excellent. Test-retest reliability was moderate to good. Test-retest reliability should be examined in further studies at a less than 2-week interval to reduce maturation bias. This instrument can be used to measure enablement after consultation in a French-speaking family practice setting.

## Background

Health promotion can be defined as the "process of enabling people to increase control over, and to improve, their health" [1]. Promoting health is at the heart of the encounter between patients and primary healthcare providers. Howie et al. [2,3] proposed that the concept of enablement represents the extent to which a patient feels enabled after a medical consultation. They advocated that this concept be used as a measure of the quality of the consultation, rather than a patient's satisfaction with it. They hypothesized that it may represent an intermediary outcome that promotes coping or self-efficacy that is linked to health and behaviour change. Two different studies conducted in Scotland [4,5] found that the impact of enablement on daily living at first consultation was highly predictive of positive changes in main complaint and well-being at 1 and 3 months, after controlling for the number of consultations among patients. In the study done by Bikker et al. [4], enablement at 3 months was predictive of changes in main complaint and well-being at 12 months. Significant positive correlations were also found between patient enablement and change in quality of life at 4 weeks and 12 weeks for patients suffering from asthma in general practices in the UK [6,7].

The Patient Enablement Instrument (PEI), which was developed to measure patient enablement after a medical consultation in primary care, showed good psychometric properties [2,3]. To date, English [2,3], Polish [8], Croatian [9], and Chinese [10] versions of the PEI have been developed. The purpose of this study was to evaluate the reliability of a French version of the PEI (PEI-Fv) in a French-speaking family practice setting.

## Methods

### Study design and setting

We carried out a reliability study of the PEI-Fv with patients attending the family medicine clinic of a regional health centre (Centre de santé et de services sociaux de Chicoutimi) in Saguenay, Québec, Canada, using a waiting-room survey immediately after their consultation (T1) with a health professional, followed by a questionnaire sent by mail 2 weeks after the consultation (T2).

### Participants and sampling

To be included in the study, a participant had to be 18 years of age or older, a regular patient of the family medicine clinic for over a year, able to read and respond to a questionnaire in French, and attending the clinic to see a physician or resident (other than the principal investigator). Patients were excluded if they had an unstable acute condition or a decompensated psychiatric condition or if they came to the clinic without a prior appointment (walk-ins) or were unable to provide informed consent. We also excluded patients who were pregnant because this condition requires special follow-up and is not a typical encounter.

In this clinical setting, patients mainly consult with physicians about issues with their chronic disease and care. In 2005, a study by Fortin et al. [11] found that overall, 9 of 10 patients in similar clinics had more than 1 chronic condition.

### Data collection

We recruited a convenience sample of patients from the waiting room of the family medicine unit between July 6 and July 16 2010. The clinic's reception staff informed each patient that a research assistant would contact them in the waiting room and gave each adult patient a yellow card to flag them as possible participants. One of 2 trained research assistants approached all potentially admissible patients. The research assistant first asked for permission to speak with the patient and then proceeded to explain the nature, objective, and procedures of the project and to review the consent form and verify the patient's eligibility to enter the study. Patients were invited to ask any questions they had. If patients agreed to participate in the study and signed a consent form, the research assistant then explained how to complete the questionnaires (the PEI-Fv and sociodemographic questions documenting age, sex, education, family income, and marital status). The patient completed the self-administered questionnaires immediately after his or her consultation with the health professional (T1). After completing the questionnaires, patients handed them to the research assistant, who stored them in a secure file.

Two weeks after the initial consultation (T2), patients received the PEI-Fv by mail with a pre-addressed, pre-stamped envelope so that they could return it once they had completed it. We adapted Dillman's method [12] to optimize responses. Two weeks later, we sent a reminder postcard to all participants who had not returned the T2 questionnaire. One week after this reminder, we contacted participants by telephone to remind them once again about returning in the completed T2 questionnaire. If the participant asked, we sent another copy of the T2 questionnaire by mail, and as a last resort, we offered to help them complete it over the telephone.

### Patient Enablement Instrument

**Figure 1:**
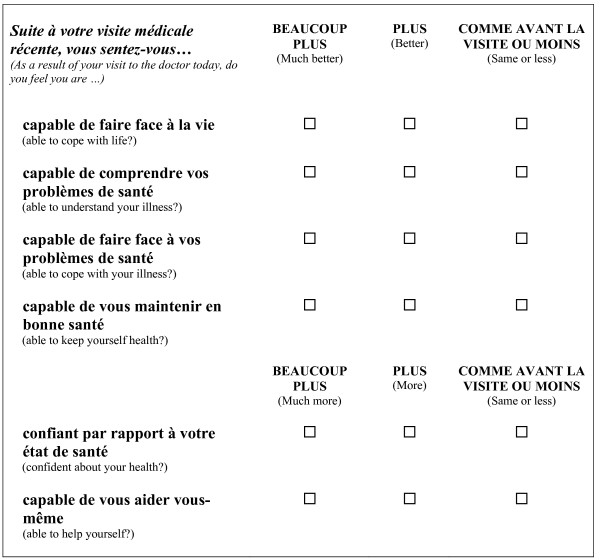
**Patient Enablement Instrument**.

The PEI (Figure 1) is composed of 6 questions, on a 3-point scale ranging from 0 to 2, corresponding to "Same or less," "Better/More," and "Much better/Much more," respectively. Results for each questionnaire may vary between 0 and 12 points (the sum of scores for each item); a score of 12 indicates that the patient experienced maximum enablement. Internal consistency of the original English version was reported as excellent (Cronbach's α = 0.93) [3]. The Chinese version showed a good test-retest reliability of 0.75, as calculated by the intra-class correlation coefficient (ICC) [10]. The rank correlation score calculated with the Consultation Satisfaction Questionnaire was 0.48 (P < 0.01) [2,3].

The rigorous translation process involved several bilingual members of the research team taking particular care to preserve the subtle meaning of items, while preserving the cultural context. First, a bilingual member of the research team translated the original version into French. A panel of bilingual team members composed of researchers from medical and nursing disciplines examined both versions and made revisions to further adapt the questionnaire into Quebec French. The translated version (PEI-Fv) was then submitted to the panel once again.

We estimated that the sample size required to calculate the ICC with 5% precision and a power of 80% with an ICC > 0.60 would be > 50 patients [13]. Calculations done according to Dillman's method [12] suggest a sample size of 100 participants.

### Analysis

We described the sample using mean ± standard deviation (SD) for continuous variables such as age and patient enablement, and proportions for categorical variables (sex, marital status, education, family income).

We calculated the internal consistency for the PEI-Fv using Cronbach's α with a 95% confidence interval (CI) and the test-retest reliability by determining the ICC (95% CI) between the 2 administrations of the questionnaire (PEI-Fv). We used the SPSS version 16.0 software for all analyses.

This study was approved by the ethics committee of the Centre de santé et de services sociaux de Chicoutimi (Québec, Canada).

## Results

The research assistants approached a total of 129 potentially admissible patients. Of these, 112 (86.8%) agreed to participate in the study; only 2 (1.8%) of these participants did not return the completed questionnaire after consultation at T1.

Table 1 summarizes participants' characteristics: mean age was 59.1 ± 16.2. Most subjects were female (67/110, 60.9%), 86.2% (94/109) of participants had at least an 8^th ^grade education, 74% (77/104) had an income ≥ CAN$20,000 and 57.8% (63/109) were married or lived with a partner. At T2, 90 (81.8%) of the 110 participants returned their completed PEI-Fv. Patients with ≥ 1 missing value on the patient enablement scale were excluded from the analysis: 2 patients were excluded from the analysis of PEI-Fv at T1 and 2 were excluded at T2.

**Table 1:** Participant characteristics (n = 110)

Characteristic	Number (n = 110)	Percentage (%)
Mean age Sex	59.1 ± 16.2	
Male	43	39.1
Female	67	60.9
Education level completed		
Grade 1-7	15	13.6
Grade 8-12	35	31.8
College or post-secondary school	33	30.0
University	26	23.6
Missing data/no response	1	0.9
Family revenue (CAN$)		
< 10,000	11	10.0
10,000-19,999	16	14.5
20,000-29,999	9	8.2
30,000-39,999	22	20.0
40,000-49,999	12	10.9
≥ 50,000	34	30.9
Missing data/no response	6	5.5
Marital status		
Married/living with partner	63	57.3
Separated/divorced	13	11.8
Widowed	17	15.5
Single	16	14.5
Missing data/no response	1	0.9

The mean (± SD) score for the PEI-Fv was 5.06 (95% CI: 4.30-5.81 ± 3.97) at T1 and 4.63 (95% CI: 3.82-5.44 ± 3.90) at T2. The French instrument showed excellent internal consistency: the Cronbach α coefficient was 0.93 (95% CI: 0.91-0.95) at T1 and 0.93 (95% CI: 0.91-0.95) at T2. The ICC for PEI-Fv measures at T1 and T2 was 0.62 (95% CI: 0.48-0.74).

## Discussion

This is the first report of the reliability of a French version of the PEI. We were able to confirm an excellent internal consistency (Cronbach α coefficient = 0.93), as described in previous studies [2,3,6,8,10,14]. The test-retest reliability was moderate to good (ICC = 0.62) [15]. The only other data available for comparison are from the Chinese validation [10] that reports an ICC of 0.75 (95% CI not provided) for a retest done 2-3 weeks after the first completion of the questionnaire. We planned on estimating the test-retest reliability in our study at 2 weeks to compare our results with those of the Chinese study. However, in the end, 27% of our sample completed the PEI-Fv T2 > 3 weeks after T1. Therefore, our weaker result could be explained by the longer delay between the completion of the PEI-Fv at T1 and at T2.

We observed that mean enablement at T2 was lower than that at T1. Lam et al. [10] also observed a decrease in mean patient enablement at T2 (4.65 at baseline and 4.22 at follow-up). The level of enablement may be maximal immediately after consultation. The difference between measures after 2 weeks could be explained by a change in the enablement level and may not be due to a difference in measure. However, we did not any identify studies documenting the evolution of the level of enablement over time after a medical consultation. Further studies should measure test-retest reliability after a shorter delay (3 or 4 days, for example). More research could also document the evolution of the level of enablement over time after a medical consultation.

In the United Kingdom, a survey of 25,994 adults done by Howie et al. [16] reported a mean PEI score of 3.1 (95% CI: 3.1-3.1). In another study [17] of patient enablement in a population of patients undergoing acupuncture (n = 52) throughout the United Kingdom, mean patient enablement was 3.62 (95% CI: 2.89-4.76). A Scottish study [5] of 323 patients reported a mean PEI score of 3.65 (95% CI not provided). The study [4] of 187 new outpatients at a Glasgow homeopathic hospital reports a mean PEI score of 3.7 (95% CI: 3.2-4.2). Finally, in another study [18] conducted by Mercer and Watt with 3,044 patients in Scotland, mean patient enablement sores were 4.0 ± 3.8 and 3.9 ± 3.5 for patients from most deprived areas and least deprived areas, respectively. Results from the studies conducted in the United Kingdom in large populations are quite constant. Results from a study in Poland [8] that used a Polish version of the instrument present comparable results: mean patient enablement was 3.65 (95% CI: 3.51-3.79) in a pilot study of 2,289 patients and 4.0 (95% CI not given) in a study of 7,924 adult consultations.

The mean score for the PEI-Fv was 5.06 (95% CI: 4.30-5.81). Our results are comparable to the results obtained for a Chinese-speaking population of 152 adults: 4.65 (95% CI: 4.21-5.10) [10]. A much higher PEI score was obtained for a Croatian population of 5,527 patients ≥ 18 years of age: the mean enablement score was 6.6 (95% CI not provided) [9].

The differences in patient enablement observed in these studies may be the result of cultural differences, as Howie et al. discuss [16]. On the other hand, in comparison with the studies conducted in the United Kingdom [2,3,5-7,16,17], the size of the current study's sample, as well as that of the Chinese sample [10], although sufficient to obtain adequate power for the objectives of the study, was relatively limited to be able to generalize a mean patient enablement score to that for a French or Chinese population. Further studies in a French-speaking setting, should measure patient enablement after consultation with larger samples. Finally, the mean score on the PEI and the length of the consultation may be linked to continuity of care [5]; however, we do not have data to examine this issue.

### Study limits

The main limitation of our study was that some patients returned their completed questionnaires more than 2 weeks after their consultation, which may have introduced a maturation bias and negatively affected the test-retest reliability of the PEI-Fv. A second limitation is that our results may not be applicable to patients on the lower end of the socioeconomic status scale because very few of the participants in our study fell into this category. In addition, our small sample size does not allow us to extrapolate the level of patient enablement to other or larger French-speaking populations; however, it did allow us to attain the objectives of our study. Notwithstanding these limits, our results confirm the reliability of our PEI-Fv.

## Conclusions

The internal consistency of our PEI-Fv was excellent. Its test-retest reliability was moderate to good. The instrument can be used to measure enablement after consultation in a French-speaking family practice setting. The test-retest reliability should be examined in further studies at shorter intervals (< 2 weeks) to reduce maturation bias. The evolution of the level of enablement over time should also be examined.

## List of abbreviations used

PEI: Patient Enablement Instrument; PEI-Fv: French version of the PEI; CI: confidence interval; ICC: intra-class correlation coefficient; SD: standard deviation.

## Competing interests

The authors declare that they have no competing interests.

## Authors' contributions

CH conceived and designed the study with MF and MEP. CH and SB drafted the manuscript. MF, MEP, and FR participated in the critical review of the manuscript. All authors gave their final approval of the version of the manuscript submitted for publication.

## Pre-publication history

The pre-publication history for this paper can be accessed here:

http://www.biomedcentral.com/1471-2296/12/71/prepub
